# Supplementary material for: Dehydrin Protein TaCOR410 Improves Drought Resistance of Wheat Through Autophagy
Source: Plants (Basel). 2025 Sep 2;14(17):2726. doi: 10.3390/plants14172726 (PMC12430722; doi:10.3390/plants14172726)
Supplement: Supplementary file 1 [file plants-14-02726-s001.zip › plants-3819609-supplementary.doc]

**Supplementary Material**

**Dehydrin protein TaCOR410 improves drought resistance of wheat through autophagy**

**Mei-Yan^1, 3^, Hua-Dong Song^2, 3^, Jia Lian-Wei^1^, Kai-Yong Fu^1^, Gang-Li^1^, Yong-Bo Li^2*^, Cheng-Li^1^***

**1 Shi He Zi University; Shihezishi, Xinjiang 832003, China.**

**2 Crop Research Institute, Shandong Academy of Agricultural Sciences, National Key Laboratory of Wheat Improvement; Jinan, Shandong 250100, China.**

**3 Shandong Luyan Seed Co．，Ltd; Jinan, Shandong 250100, China.**

[yanmei1029@163.com](mailto:yanmei1029@163.com) (M.-Y); shd9227@sina.com (H.-D.S); [m18386191910@163.com](mailto:m18386191910@163.com) (J.-L.W.); fukaiyong@stu.shzu.edu.cn (K.-Y. F.); ligang@xjshzu.com (G.-L).

*** Correspondence: lyb920327@sina.com (Y.-B.L.); licheng@shzu.edu.cn (Ch.-L).**

**Figure S1 Effect of PEG-8000-induced stress simulating drought on autophagy related genes (*ATGs*)’ expression.** Effect of 20% PEG-8000 treatment for 3, 6, 12, 24 and 48 h on leaf mRNA levels of *TaATGs* (*TaATG3*, *4*, *5*, *6*, *7*, *8*). *ɑ-Tubulin* was used as an internal reference and the relative expression was calculated using the 2^_ΔΔT^ method. Control: treatment with water. Bars represent the mean ± SD of three independent experiments. * and **indicate significant differences at p < 0.05 and p < 0.01, respectively, according to the Student’s *t*-test.

**Table S1. The primers used in this study**

| Primer name | Sequence (5’-3’) |
| --- | --- |
| ATG3QRTf | CCCTCCGATAAGCAGTTC |
| ATG3QRTr | TTTCACCAGCACCAAAGTA |
| ATG4QRTf | GAAAGCCCGCACAGAGTC |
| ATG4QRTr | ACCCGAGACCACATAGAGC |
| ATG5QRTf | GCTGCCTTCATCATAACTGG |
| ATG5QRTr | ATCGCATCTTCTAAATACTCG |
| ATG6QRTf | CAAACTTATGGCAAACTCG |
| ATG6QRTr | CAAACTTATGGCAAACTCG |
| ATG7QRTf | TGCCTCACTGGTGCTTAG |
| ATG7QRTr | CAATCCTTGAGTTGCCTTA |
| ATG8QRTf | AGGCTGATAAGTCTGATGTCC |
| ATG8QRTr | CGTCCTCGTCCTTGTTTT |
| COR410QRTf | AGAGGAGCACCCAGTCGTAC |
| COR410QRTr | GCTCTTCCACGGAGACCTT |
| ɑ-TublinF | AACTTCGCCCGTGGTCAT |
| ɑ-TublinR | CAGCGTTGAATACAAGGAATC |
| GFPrnaiF | ATGGTGAGCAAGGGCGAGG |
| GFPrnaiR | AGAAGATGGTGCGCTCCTG |
| ATG5rnaiF | ATGTGATGAATATGTCCCAG |
| ATG5rnaiR | AGGTACCCTGCATGGTTTGAC |
| ATG7rnaiF | GAAGCCATCCAGATGGAAAT |
| ATG7rnaiR | CATGACAAGGTAACTGTCATG |
| COR410-AIMf | ACCCTCGCCTCCAAGGCGCACC |
| COR 410-AIMr | GGTGCGCCTTGGAGGCGAGGGT |

*ATG* (*3*, *4*….) QRT primers are used for Quantitative real-time reverse transcription-PCR (qRT-PCR) of *autophagy related genes* (*ATGs*). *ATG5*, *7* RNAi primers are used for amplifying the interference sequence of *ATG5, 7*; COR410QRT primers are used for amplifying the interference sequence of *TaCOR410* and *GFP* RNAi primers are used for the control. COR410-AIM primers are used for amplifying AIM motif sequence.
